# Comparison of geometric morphometric outline methods in the discrimination of age-related differences in feather shape

**DOI:** 10.1186/1742-9994-3-15

**Published:** 2006-09-15

**Authors:** H David Sheets, Kristen M Covino, Joanna M Panasiewicz, Sara R Morris

**Affiliations:** 1 Department of Physics, Canisius College, 2001 Main St., Buffalo, NY 14208, USA; 2 Department of Biology, Canisius College, 2001 Main St., Buffalo, NY 14208, USA

## Abstract

**Background:**

Geometric morphometric methods of capturing information about curves or outlines of organismal structures may be used in conjunction with canonical variates analysis (CVA) to assign specimens to groups or populations based on their shapes. This methodological paper examines approaches to optimizing the classification of specimens based on their outlines. This study examines the performance of four approaches to the mathematical representation of outlines and two different approaches to curve measurement as applied to a collection of feather outlines. A new approach to the dimension reduction necessary to carry out a CVA on this type of outline data with modest sample sizes is also presented, and its performance is compared to two other approaches to dimension reduction.

**Results:**

Two semi-landmark-based methods, bending energy alignment and perpendicular projection, are shown to produce roughly equal rates of classification, as do elliptical Fourier methods and the extended eigenshape method of outline measurement. Rates of classification were not highly dependent on the number of points used to represent a curve or the manner in which those points were acquired. The new approach to dimensionality reduction, which utilizes a variable number of principal component (PC) axes, produced higher cross-validation assignment rates than either the standard approach of using a fixed number of PC axes or a partial least squares method.

**Conclusion:**

Classification of specimens based on feather shape was not highly dependent of the details of the method used to capture shape information. The choice of dimensionality reduction approach was more of a factor, and the cross validation rate of assignment may be optimized using the variable number of PC axes method presented herein.

## Background

Quantitative morphometric methods have long been used to classify organisms. Discriminant function analysis (DFA) or canonical variates analysis (CVA) are often used to support the identification of distinct species, particularly in fossil lineages [1-4] and alternative statistical approaches to the classification of specimens based on maximum likelihood methods have also been developed [5]. The explicit assumption is that a low rate of misclassification of individuals from two populations provides evidence for genetic differences [6].

Traditional quantitative morphometrics have made use of a variety of lengths, widths, angles, and ratios to capture information about shape. Geometric morphometric approaches to shape have focused on complete, uniform measurement of shape, retaining all geometric information throughout the analysis. Within this context, measurement of curves or outlines poses some challenges, since mathematically curves are infinite sets of points. The use of multivariate statistical methods (specifically canonical variates analysis, CVA, a multiple group form of discriminant analysis) to classify specimens into groups requires that the curves or outlines on the specimens be represented by a limited number of measured variables. The linear CVA requires a matrix inversion of the pooled covariance matrix requiring more specimens than the sum of the number of groups and measurements per specimen. Classification of specimens based on outlines thus poses a challenge, in that accurate representation of a curve requires many measurements accurately, but this increase in parameters dramatically increases the sample sizes necessary to carry out the CVA.

A variety of geometric morphometric approaches to curves have been used, but comprehensive assessment of their performance in a CVA has been limited. Curves have been represented by mathematical functions [3,7-11] or by a limited number of discrete points [12-15]. One of the newer innovations is the class of semi-landmark methods that incorporate information about curves into the landmark-based formalism [14-18]. This approach allows for the combination of information about discrete homologous points (i.e., the landmarks) with information about curves into a single analysis. There have been studies comparing the effectiveness of outline-based methods to traditional measurements [3] or outlines to landmark-based methods [19], and there have been comparisons among semi-landmark-based methods [4] but apparently no direct comparison of different outline-based methods.

Applying DFA or CVA to outline data requires first digitizing the structure, then aligning the structures to compensate for any arbitrary decision in the digitizing process, and finally extracting a discriminant function or a set of canonical variate axes from the data. The impact of alternative approaches to digitizing and aligning the structure on the detection of differences in mean shape or discrimination among groups has not been established. Among the methods for digitizing the curves are (1) template- or fan-based methods, in which a set of points is defined *a priori *by some rule (i.e., equal angles between all radii of a circle, with the points to be digitized being located at the intersection of the radii and the outline curve); (2) manual tracing of curves, in which points are selected by eye as the curve is traced; and (3) automated curve tracing, in which software is used to detect differences in color or brightness to delimit the curve. Additionally, semi-landmark methods (bending energy minimization or perpendicular projection), elliptical Fourier analysis, and extended eigenshape analysis approach the alignment process differently. The interaction between digitization and alignment may affect the ability to discriminate among shapes.

Once the data are collected and aligned, yet another potential methodological question must be addressed because methods like CVA and DFA require that there be more specimens than variables. The linear CVA requires a matrix inversion of the pooled within-group variance-covariance matrix, requiring that it be of full rank, which in turn requires more measured specimens than the sum of measurements per specimen and groups. If this condition is not met, there are more degrees of freedom in the measurements than in the specimens. The quadratic form of CVA requires independently estimated covariance matrices for each group and thus places even greater demands on the data. Fortunately, the linear method is quite robust and often outperforms the quadratic method even when the covariance matrices are unequal [20,21].

The use of outline methods thus poses difficulties for CVA, both due to the large number of semi-landmarks needed per specimen to describe outlines and due to the representation of semi-landmark points by two coordinates (*x*- and *y*-) when there is only one degree of freedom per point. While points along the curve are originally measured as a pair of Cartesian coordinates, only one degree of freedom remains after the semi-landmark alignment procedure is used. Principal components analysis (PCA) may be used to reduce the dimensionality of the data by analyzing a limited number of PC scores of the specimens instead of the original data. This reduction poses a need for an objective criterion to determine the number of PC scores used. The simplest approach is to use as many PC axes as possible, given the degrees of freedom in the data, i.e., retaining all PC axes with non-zero eigenvalues.

In many studies, including ours, the rate of correct classification of specimens is a primary concern. Thus, we would suggest that optimization of the classification rate of the subsequent CVA be the objective criterion for determining the number of PC scores used in dimensionality reduction. There are two approaches to estimating the rate of correct assignments: resubstitution and cross-validation. The resubstitution estimator (the rate of correct assignments of specimens used to form the CVA axes) is known to be biased upwards [6], since this estimate of the success rate is based on the same data that is used to form the discriminant function. A better estimate of the classification rate may be obtained through cross-validation, in which one or more specimens are left out of the "training set" used to form the discriminant function [6]. The specimens left out of the training set can then be assigned to groups based on the discriminant function, with less upward bias than in the resubstitution rate. The use of large numbers of PC axes in the CVA may yield high rates of correct assignments based on the resubstitution estimator but substantially lower cross-validation rates due to overfitting the discriminant axes to the data, with a subsequent loss in generality (see discussion of overfitting in [22]). Reducing the number of PC axes used in the analysis may result in lower resubstitution rates, but higher cross-validation rates.

In addition to methods of dimensionality reduction based on PCA, a method based on a partial least square regression technique has also been proposed [23]. In this method, the covariance matrix between the measurements and a matrix of classification codes (with one column per group or class) is calculated. This covariance matrix is then decomposed using a singular value decomposition (SVD), which yields SVD axes that are linear combinations of the original measurements that show the greatest covariation with the classification variables. In the approach used by Kemsley [23], there is one SVD axis generated per class in the CVA. One then carries out the CVA on the scores of the specimens along these SVD axes. Kemsley reported that this method produced higher rates of correct classification (both resubstitution and cross-validation) than PCA-based dimension reduction.

Since the statistic of interest in many studies is the rate of correct assignments based on cross-validation, an alternative approach is to choose the number of PC axes that result in the highest cross-validation rate of correct assignments. This may be done by calculating cross-validation rates for a wide range of differing numbers of PC axes and using the number of PC axes that optimizes the cross validation assignment rates. The bootstrapping approach outlined by Solow [6] is then used to determine a confidence interval on the cross-validation assignment rate, by resampling the data (with replacement) and then carrying out the entire CVA analysis, including the determination of the number of PC axes to use, on the bootstrapped data set. The distribution of optimal cross-validation assignment rates over the bootstrap sets can then be used to determine confidence intervals on the cross-validation rate of classification. Our approach differs from others proposed to date [24,25] in using the cross-validation rate of assignment as the objective criteria for the number of dimensions to use. These methods determine the number of PC axes to use in carrying out the CVA based on examination of the properties of the pooled covariance matrices themselves, rather than the end results [24,25].

The goal of this study is methodological, focusing on the performance of different measurement and data acquisition procedures in classifying specimens based on outlines using canonical variates analysis. In this study, we compare the performance of two semi-landmark alignment methods (perpendicular projection [PP] and bending energy minimization [BEM]), elliptical Fourier analysis (EFA) and the extended eigenshape method to classify specimens. Additionally, we compare several approaches to data acquisition, manual curve tracing, template-based digitization, and automatic edge detection, as well as assessing the dependence of these methods on the number of points used in the analysis. We used as a test case the rectrices (tail feathers) of a single species of bird, the ovenbird (*Seiurus aurocapilla*), belonging to different age categories. Age-related differences in feather shape are common in many species and provide a good data set for a methodological study because there is a known, but subtle, difference in tail shapes between birds that are under a year old and birds that are more than a year old. In the field, experienced bird banders are often able to discriminate between age categories of birds by visual inspection of rectrix (tail feather) tip shape (e.g., [26-29]). Traditional morphometric measurements – rectrix tip angle [30-32] and width of rectrices [33]– also have been used to distinguish between birds in their first year and older birds. Among ovenbirds, rectrix tip shape has been documented to be more truncate among adult birds than young birds [32,34]. Additionally, in this study, the age of specimens could be determined independently of feather shape based on dissection of these previously collected specimens. Moreover, feathers present a challenge to digitization, in that the edge of the feather is occasionally difficult to distinguish. Ontogenetic differences in ovenbird feathers thus present a system with a known variation in shape, but with some challenges to successful discrimination of shape.

## Results

Automatic curve tracing did not work well with the specimens in this study. Since the automated curve tracing actually required manually tracing the feather outline for our data set to get reliable detection of the outline edge, due to the irregular edges of the feathers, and did not appear to offer higher repeatability (Table 1), we did not attempt to carry out automated curve tracing on the remaining specimens. When bending energy minimization (BEM) was used with data acquired using a fan, there was a lower variation within repeated measures than when the data was acquired using curve tracing (Table 1). In contrast, perpendicular projection (PP) alignment yielded very similar estimates of variation in data acquired using fans and curve tracing. The ratio of the variation in repeated measurements of a single specimen to the total population variance using curve tracing was 0.077 for BEM and 0.020 for PP, while this ratio was 0.164 for BEM of fan-digitized data and 0.035 for PP of fan-digitized data. Manual curve tracing also took less time than the fan-based method to digitize feathers. The average digitizing time using curve tracing was approximately 4 minutes, whereas the same operation using a fan averaged roughly 6.5 minutes.

**Table 1:** Repeated measures variance under different measurement protocols using a single feather

**Data acquisition**	**Data processing**	**Variance**	**95% confidence interval for variance**
Automated	Bending energy	0.000594	0.000287 – 0.000780
Curve tracing	Bending energy	0.000213	0.000107 – 0.000295
Fan	Bending energy	0.000114	0.000057 – 0.000144
Automated	Perpendicular projection	0.000046	0.000027 – 0.000053
Curve tracing	Perpendicular projection	0.000011	0.000008 – 0.000011
Fan	Perpendicular projection	0.000009	0.000007 – 0.000009

Each protocol used 82 points around the periphery of the feather. A single feather was digitized 10 times. The confidence interval was determined using bootstrap resampling with replacement. The bending energy minimization always produced higher variance estimates than the perpendicular projection method.

When a fixed number of PC axes (40, to allow for slightly more specimens than variables when carrying out cross validation calculations) were used to reduce the dimensions of the data prior to the CVA, there was strong evidence of over-fitting (Table 2). The resubstitution rate of correct assignments was 100% for all methods using 40 PC axes. The cross validation rate of correct assignment varied from 58.7% to 78.3% for 40 PCA axes. The PLS method produced lower resubstitution rates (76.1% to 89.1%) but typically slightly higher cross validation rates (54.4 to 82.6%). The variable PCA method introduced here produced intermediate resubstitution rates (82.6% to 93.5%) but consistently high cross-validation rates (69.6 to 89.1%) and the difference between resubstitution and cross validation rates was greatly reduced.

**Table 2:** Classification rates produced by a CVA after each of the three dimensionality reducing approaches considered

		**40 PC axes**		**PLS**		**Variable PC axes**		
**Data acquisition**	**Data processing**	**RS rate**	**CV rate**	**RS rate**	**CV rate**	**# of axes used**	**RS rate**	**CV rate**

Curve Tracing	Bending energy	100	69.6	76.1	73.9	9	82.6	87.0
Curve Tracing	Perpendicular projection	100	69.6	87.0	73.9	13	89.1	84.8
Curve Tracing	Elliptical Fourier analysis	100	58.7	87.0	65.2	12	87.0	73.9
Curve Tracing	Eigenshape analysis	100	65.2	82.6	54.4	24	93.5	69.6
Fan	Bending energy	100	65.2	89.1	82.6	7	89.1	89.1
Fan	Perpendicular projection	100	78.3	89.1	82.6	7	93.5	89.1

In each case, the canonical variate axes were significant using a Wilk's lambda test at p < 0.005 or better. Resubstitution (RS) were higher than cross-validation (CV) rates for the fixed number of principal component (PC) axes and partial least squares (PLS) methods, and closer to equal for the variable PC axes method. All rates are listed as percentages.

The ability to correctly discriminate among specimens from the two age groups did not depend on the approach to digitization or on the alignment method (Table 2). The optimal rate of cross-validation assignment was remarkably consistent for the different approaches to semi-landmarks. The differences in cross-validation assignment rates (± 2.2 %) observed among the semi-landmark methods amounted to assignment of a single specimen. The cross-validation rate for EFA data was not as high, although the confidence interval for the EFA-based assignment rate was very similar to that derived from the semi-landmark methods. The estimated cross-validation rate for the eigenshape data was lower still. However, the bootstrap-derived estimate of the 95% confidence interval on the cross-validation rate for eigenshape data indicated that the observed rate was at the lower edge of the confidence interval (Table 3). The confidence interval itself was virtually identical to that obtained using the other methods.

**Table 3:** Bootstrap estimates of the cross-validation rates of CVA assignments : Variable number of PC axes method

			**Cross-validation assignment rate (%)**	
			
**Data acquisition**	**Data processing**	**# of PC axes**	**Observed**	**95% confidence interval (derived from bootstrap)**
Curve tracing	Bending energy	9	87.0	69.6 – 95.7
Fan	Bending energy	7	89.1	76.1 – 95.7
Curve tracing	Perpendicular projection	13	84.8	76.1 – 97.8
Fan	Perpendicular projection	7	89.1	78.3 – 97.8
Curve tracing	Elliptical Fourier analysis	12	73.9	63.0 – 93.5
Curve tracing	Eigenshape analysis	24	69.6	67.4 – 95.7

Each method of outline processing shown used 82 points around the periphery of the feather. Rates of cross-validation assignment based on canonical variates analysis (CVA) were similar for all methods, given the overlapping 95% confidence intervals. The number of principal component (PC) axes used to optimize the cross-validation assignment rate varied slightly over the different methods.

The number of points included in the analysis had little impact on the results, although we should note that we could not readily vary the number of points using fan-based digitization. The error in the estimated length of the curve based on a reduced number of points seemed to be quite low over the range of points used in this study (Table 4). The ability to discriminate similarly showed very little dependence on the number of semi-landmarks used (Table 5), with the only differences appearing at 20 and 30 semi-landmarks when using curve tracing and the BEM.

**Table 4:** Error in the length of the curve as a function of the number of points used

**# of Points**	**Mean error**	**Largest observed error**
120	0.67%	1.14%
82	0.86%	1.54%
60	1.02%	1.67%
41	1.46%	2.43%
30	1.87%	2.95%
20	2.46%	3.55%

Initial digitization involved recording >200 arbitrarily space points along the periphery of the feather. Reduction of these points to a fixed number of equally spaced points (20 to 120) along the outline inevitably produced error. This error is expressed as a percentage of the total original length of the outline. The mean error is the average over all specimens in the study; the largest observed error is the largest over all specimens. Error increases as the number of points used to approximate the curve decreases.

**Table 5:** CVA results using variable number of semi-landmark points used.

				**Cross-validation assignment rate (%)**	
				
**Data acquisition**	**Data processing**	**# of points used**	**# of PC axes**	**Observed**	**95% confidence interval**
Curve tracing	Bending energy	20	18	67.4	67.4 – 93.5
Curve tracing	Bending energy	30	18	78.3	67.4 – 93.5
Curve tracing	Bending energy	41	10	84.8	73.9 – 95.7
Curve tracing	Bending energy	60	10	84.8	69.6 – 95.7
Curve tracing	Bending energy	82	9	87.0	69.6 – 95.7
Curve tracing	Bending energy	120	9	87.0	67.4 – 95.7
Curve tracing	Perpendicular projection	20	18	84.8	76.1 – 97.8
Curve tracing	Perpendicular projection	30	10	82.6	76.1 – 95.7
Curve tracing	Perpendicular projection	41	11	84.8	71.7 – 95.7
Curve tracing	Perpendicular projection	60	13	87.0	73.9 – 97.8
Curve tracing	Perpendicular projection	82	13	84.8	76.1 – 97.8
Curve tracing	Perpendicular projection	120	12	84.8	76.1 – 95.7
Curve tracing	Elliptical Fourier analysis	41	10	84.8	65.2 – 93.5
Curve tracing	Elliptical Fourier analysis	82	12	73.9	63.0 – 93.5
Fan	Bending energy	41	6	89.1	73.9 – 95.7
Fan	Bending energy	82	7	89.1	76.1 – 95.7
Fan	Perpendicular projection	41	9	87.0	73.9 – 97.8
Fan	Perpendicular projection	82	7	89.1	78.3 – 97.8

The rate of correct cross validation assignment based on the canonical variates analysis (CVA) was not highly dependent on the number of points used to represent the curve. The number of principal component (PC) axes used to optimize the cross-validation assignment rate varied with the data acquisition and processing methods and the number of points on the outline.

An experienced bird bander correctly classified 38 (82.6%, n = 46) of all individual rectrices, similar to the percentage correctly assigned by the geometric morphometric methods. When the class of indeterminate feathers was removed, the proportion correctly classified increased to 90.3% (28 of 31). The more traditional method of using all tail feathers resulted in a classification rate of 93.5% (43 of 46), which increased to 97.4% (37 of 38) without indeterminate specimens.

## Discussion

Discrimination between age classes based on shape is robust to changes in data acquisition methods, semi-landmark alignment criteria, and method of shape analysis (semi-landmarks, EFA, and eigenshape). We find no evidence for the superiority of curve-tracing or fan-based methods of data acquisition for semi-landmark alignment for the specific example of feather outlines. However, the average time to digitize feathers was lower for curve tracing than fan-based methods, and curve tracing also allowed for easy variation in the number of points used in the final analysis. Based on these results, we would recommend curve tracing over the use of fans or templates.

Methods of semi-landmark alignment can be directly compared with respect to repeatability, whereas it does not seem legitimate to directly compare numerical results of partial Procrustes distances or summed squared distances based on BEM with those based on PP. Although the specimens may be in the same linear tangent space, they were not projected into that space in exactly the same manner. Since a single curve projects into different locations under the two alignment schemes, the difference in the two methods is not linear with respect to distances measured in the two groups. Thus, rather than using raw variance measures, ratios of variance of repeated measures of a single specimen to the whole population variance were used to compare BEM and PP. BEM produced a larger ratio of variation in repeated measures of a single specimen to the variation in the entire sample than PP whether using a fan or curve tracing. Curve tracing produced a lower ratio than fan-based digitization. Since the total variance in the sample is the sum of biological variance and measurement error, it appears that the BEM results in higher variation in measurements than PP for this data set.

The method of shape analysis–specifically whether the analysis was based on semi-landmark methods, EFA, or extended eigenshape analysis–had very little effect on the results. The different methods produced very similar rates of successful discrimination, based on the bootstrap estimates of the 95% confidence interval of the cross-validation rate of correct assignments. The semi-landmark methods did provide slightly higher observed cross-validation rates, perhaps due to the semi-landmark alignment or to the use of centroid size rather than outline length as a measure of size. It should be noted that the semi-landmark methods do incorporate "sliding" of semi-landmark points along the estimated curves, and thus the semi-landmark processing methods are not related to one another, or to the Fourier and eigenshape methods, by a simple linear transformation of variables. It is reassuring to note that the method of capturing the outline did not strongly affect the results of the analysis, indicating that any of the methods could be reasonably used to study outlines. However, the semi-landmark methods do allow ready incorporation of landmark data points not along a curve into the same analysis as outline data, which may prove advantageous in studies where interior structure is of importance. While this study did not incorporate landmark data in the CVA, an earlier ontogenetic study [35] indicated that the information carried by semi-landmarks and semi-landmarks are comparable.

The approach to dimensionality reduction presented here yielded higher rates of cross-validation assignment than the simple approach of using a number of PC axes equal to the degrees of freedom in the system, or the PLS method. In the original discussion of the randomization test to determine the range of classification probabilities, Solow [6] made use of backward variable selection to determine which variables to include in the discriminant function. This type of automatic variable selection will probably reduce the types of over-fitting of the discriminant function that we have observed (our CVA algorithm did not include such a variable reduction feature). We directly maximized the cross-validation assignment rate, rather than applying indirect approaches such as backwards or forwards variable selection, or determination of the number of axes to use based on the characteristics of the pooled variance-covariance matrix employed. Variable selection might produce further optimization of these rates; however, cross-validation rates approach the resubstitution rates for our data, indicating a balance between generality and precision. The Curse of Dimensionality [36] also appears in genetic data [37,38], which suffers from the same difficulty of sample size relative to the number of variables as appears in semi-landmark data, so perhaps these approaches will prove useful in other contexts.

## Conclusion

While a clear statement of the superiority of one method of outline analysis over the others would make for a resounding conclusion, this was not the case for this study. The general consistency of our results and the characteristics of CVA indicate the promise offered by semi-landmark methods of integrating information about outlines or curves with landmark points. Semi-landmark methods were as effective in capturing the outlines of the feathers as the more established Fourier and eigenshape approaches. The variable number of PC axes method of dimensionality reduction does serve to optimize the cross-validation rates of correct assignment when used in conjunction with outline data. Furthermore, this method produces higher cross validation rates than either a fixed, maximal number of PC axes, or the partial least squares method. Manual curve tracing was the preferred method of digitization, in that it was as reproducible as other methods, offered flexibility in the number of semi-landmarks used, and proved to be slightly faster than template-based digitization.

## Methods

### Study specimens and human assessment

Forty-six known age ovenbird specimens from the Canisius College Vertebrate Collections were utilized in this study. Age determination of each specimen occurred by dissection and examination of skull pneumatization. Incomplete skull pneumatization indicates a young (hatch-year) bird, while adults have a fully pneumatized skull. In addition, a bird bander with extensive experience with ovenbirds (SRM) assigned each individual to an age category using two different criteria. The first characterization involved examination of the right, fifth rectrix, the feather that was digitized for this study. Typically, bird banders designate each individual as young, adult, or indeterminate. Indeterminate feathers normally would not have been assigned an age category in the field, but in this study were further subdivided into either adult or young categories to match the two categories used by the CVA. Additionally, the bander examined all the feathers of the tail, which is what is typically done in the field, using the same initial classifications of young, adult, or indeterminate (subsequently subdivided into young and adult). The right fifth rectrix was removed from each specimen, and the upper surface was scanned using a flat-bed scanner. Prior to scanning, the barbs and barbules of each feather were arranged in the typical interlocking manner, producing intact vanes. A blue background provided contrast and a ruler provided scaling information in each scanned image.

### Data acquisition and approaches to digitizing curves

Three different approaches to the digitization of points along curves were examined during this study. The simplest approach was to measure points spaced on the curve in an arbitrary manner, obtaining a dense sampling of points around the curve, and then use interpolation methods to reduce this set of points to some desired, fixed number of equally-spaced points [3,8,9,12]. We refer to this approach as manual curve tracing. The second approach was to use a template (hereafter referred to as a fan) on the digital image that provided guidelines to delineate equally linearly-spaced, or equally angularly-spaced, points along the curve [35,39]. The final approach was to use an automated approach to digitization, in which a computer is used to detect changes in an image (color or contrast levels), which indicates the edge of the specimen [11,40]. After the automated detection and tracing of the edge, the number of points used was then reduced in the same manner as in the manual curve-tracing approach.

**Figure 1:**
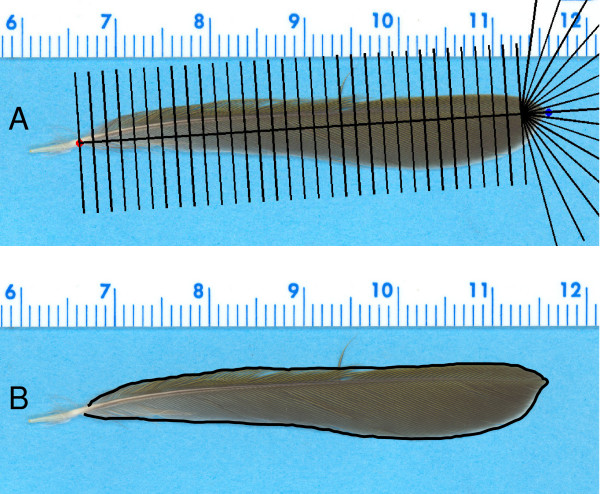
**Digitization using a fan template (A) and manual curve tracing (B) of the same feather**. When using a fan template, points were placed at the intersection of the fan and the feather margin. Manual curve tracing involved placing ≥ 200 points around the periphery to capture the outline.

Using the tpsDig program [41] for manual curve tracing (using the "draw curves" mode), we digitized at least 200 closely spaced points around the periphery of the feathers (Fig. 1). Each tracing began at the most proximal point where the barbs of the leading edge of the feather met the rachis and ended at the analogous point on the trailing edge of the feather. This set of points was then used to generate data sets of a chosen number of points (from 20 to 120) around the periphery of the feather. Linear interpolation was used to obtain equally spaced points from the originally measured, irregularly spaced points. To determine the number of points necessary to describe the curve, we followed MacLeod's [12] suggestion of using the error in the length of the curve. This error was calculated as the percentage change in the curve length caused by the interpolation. The points obtained this way were then analyzed using semi-landmark methods, Fourier methods, and extended eigenshape methods.

We used a software tool, MakeFan6 [42], to plot fans on the image of each feather, digitizing points at the intersection of the curve and the lines of the fan. When constructing the fan, we anchored the ends of the fan at the tip of the feather and at the proximal end of the feather vane where it met the rachis (Fig. 1). A variety of different fans or "combs" allow for either equal linear or equal angular spacing to be used, with anchoring based on two to four landmark points. The fan used in our measurement protocol had 82 total points, 16 of which were in a semi-circular pattern near the tip with equal angular spacing, and the remaining 66 were evenly linearly-spaced along the length of the feather. We also reduced this original set of 82 points to a 41-point data set by omitting every other point in the fan. Points obtained using the fan method were analyzed only as semi-landmarks because the unequal point spacing of the fan makes these points inappropriate for use with Fourier and eigenshape methods.

Using the automatic edge detection option in tpsDig, we automated the digitization. The uneven edges of the feather limited the effectiveness of automatic edge detection, making it necessary to trace the outline of the feather using the pen tool in tpsDig prior to automatic edge detection. This additional manual procedure removed many of the advantages of the automatic edge detection method, leaving little practical difference in the operation of the edge detection method and the manual curve-tracing method. These feather data thus did not provide a good test set for the comparison of automatic versus manual curve tracing, except to the extent that other features of interest may similarly require considerable image enhancement.

### Semi-landmark processing

Once a set of points around the curve was digitized, these semi-landmarks were processed using one of two alignment algorithms intended to reduce effects of the arbitrary selection of a limited number of points to represent the entire outline. Two different approaches, bending energy minimization (BEM; see [15,17]) and perpendicular projection (PP; similar to that used by [14]), were used to align the semi-landmarks along the curves. The initial stages of semi-landmark alignment were the same for the two methods: the landmarks and semi-landmarks were first submitted to a generalized Procrustes analysis (GPA). This standard landmark-based morphometric method removes all differences between the specimens that can be attributed to the location of the specimen, to differences in orientation (or rotation), and to scale. In this study, a partial Procrustes superimposition [43,44] was used, which fixes the centroid size (the square root of the summed squared distances of landmarks about the centroid) at 1. The GPA iteratively estimated a mean form and aligned all specimens on it.

**Figure 2:**
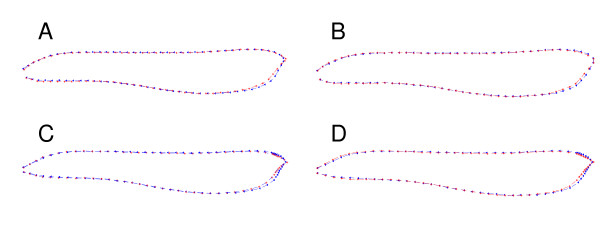
**Mean shapes under different semi-landmark methods**. The mean of young specimens is shown is represented by the blue outline, and the mean of adults is represented by the red outline, each used 82 points. (A) Bending energy alignment using curve tracing, (B) perpendicular projection using curve tracing, (C) bending energy alignment using a fan, and (D) perpendicular projection using a fan.

After initial estimation of the mean shape of the specimens (Fig. 2), semi-landmark alignment was used to select a set of points used to represent the information contained in the homologous curves. Both methods started by estimating the tangent to the curve at each measured semi-landmark point. The landmarks were then moved along the tangent either to produce the smoothest possible deformation from the reference form (BEM; [15-17,45]) or to remove all variation tangent to the curve (PP; see [14]).

BEM alignment was carried out using the tpsRelwarp program (version 1.39, [41]). The positions of the semi-landmarks (along contours) of each feather were allowed to slide along the direction parallel to the contours to minimize the bending energy necessary to produce the change in the contour relative to the reference form (the GPA-estimated mean form). This method is equivalent to the conservative assumption that the contour of a particular specimen is the result of the smoothest possible deformation of the reference form [15]. The reference form was then re-estimated after sliding the semi-landmarks of each specimen. This procedure was iterated until a stable mean form was obtained. As N. MacLeod (personal communication) notes, this procedure may result in a geometric construction (the GPA reference form) having substantial influence on the analysis, an effect which might be checked by using a fixed biological specimen as the reference form, rather than utilizing an iterated GPA mean shape. We used a GPA mean reference because other choices of reference can pose other, potentially more serious, problems [46].

Semi-landmark alignment based on the PP method was carried out using the SemiLand6 program [42]. The components of the differences in semi-landmark positions between the reference form and the target form that are tangent to the curve were mathematically removed. This procedure resulted in an alignment of the semi-landmarks on the target form along lines perpendicular to the curve passing through corresponding semi-landmarks on the reference form (see [14]). As long as the contours lack abrupt curvature changes relative to semi-landmark spacing, this criterion minimizes the distance between the semi-landmarks on the target and the reference.

Once the semi-landmarks were aligned under one of the two criteria, they were treated as points in a landmark-based analysis carried out in the linear tangent space to the underlying curved shape space [44,47]. Statistical procedures, however, must account for the reduction of one degree of freedom per semi-landmark lost in the semi-landmark alignment procedure.

### Elliptical Fourier analysis

Data gathered using the manual curve-tracing approach was analyzed using elliptical Fourier analysis (EFA), a fairly standard approach to outline data [3,8,11,48]. In this particular implementation of EFA, a set of equally linearly-spaced points around the outline was formed from the curve-traced data and the centroid position was set to the origin. The Fourier transforms of the *x*- and *y*- coordinates of these points were obtained. To standardize specimen size and orientation, the length of the ellipse formed by the first harmonic was scaled to one, and this ellipse was oriented along the *x*-axis.

### Standard eigenshape analysis

The standard eigenshape analysis of shape [12,13] started with a series of equally linearly-spaced points around the closed outline, starting at a fixed landmark. This set of Cartesian coordinate points was then converted into the φ shape function, which is the net angular deviation between the chords connecting adjacent landmark points around the outline. The φ shape function may be thought of as a series of turning angles that specifies the directional changes necessary to move around the outline from one point to the next, resulting in one angular value per point around the outline. Given the spacing between the points around the outline, it was possible to use this set of angles to calculate the relative Cartesian coordinates of the points on the outline. It is common to convert φ to a normalized form φ* by subtracting the net angular change expected for a circle of the same size. This approach to shape measurement removed differences attributable to translation and rotation by measuring all angles relative to the orientation of the adjacent chord between points on the outline, so there is no information remaining about absolute orientation or starting location in φ*. Eigenshape analysis removes the effects of size by spacing outline points equally around the outline, rather than standardizing centroid size.

A singular value decomposition of the variance-covariance matrix of the φ* values [49,50] was used to produce a set of axes that summarize the greatest variation along an ordered number of axes, as in a conventional PCA. The set of scores for specimens along these axes was then submitted to further analysis. The use of the term eigenshape data for this specific type of φ*-based outline data was in keeping with established literature [12,49], although we note that eigenvector decompositions are common in many other contexts, including PCA.

### Variation in repeated measurements of a single specimen

To estimate digitizing error, a single image of a single specimen was digitized ten times by a single operator. Each of the ten images was then analyzed using each of the three measurement methods and subjected to both methods of semi-landmark alignment. The summed-squared partial Procrustes distances about the mean shape divided by the number of specimens minus one was used as a measure of the variation in the measurements. Resampling with replacement was used to estimate a confidence interval for this variation. Data aligned by BEM and data aligned by PP were not directly compared to one another because of the difference in semi-landmark alignment criteria. Instead of direct comparisons of variation, we compared the ratio of the variation in repeated measures of a single specimen to the variation in the entire sample (all adult and young).

### Discriminating between two groups based on shape

To discriminate between age-classes by shape, we used CVA. For the semi-landmark data, partial warp and uniform component scores based on the thin-plate spline decomposition were used [43,51,52]. Partial warps are a linear transformation of the original coordinates and thus will not affect the performance of the linear canonical variates axes. The EFA and eigenshape data were submitted to the CVA without additional processing. CVA requires a matrix inversion of the pooled within-group variance-covariance matrix. PCA was used to produce the necessary degree of dimensionality reduction. To determine the number of PC axes to retain, we calculated the cross-validation rate achieved using from 1 to *df *(the number of degrees of freedom in the system) PC axes and used the number of axes that produced the highest cross-validation rate.

A simple cross-validation protocol was used throughout the study. The cross validation assignment rate was determined by sequentially selecting a single specimen at a time as the test data. The CVA was carried out on the training set and the resulting CV axis was used to classify the test data. The success rate over all specimens forms the estimate of the cross validation rate. Each of 46 specimens was used sequentially as the cross validation specimen. Thus, there were 45 specimens in the training set and 1 specimen in the test set at time, producing a total of 46 possible cross-validation sets available under this protocol, More complex methods of forming test sets in cross validation are available, but the simple approach used here appears to yield reasonable and consistent results. The bootstrapping approach outlined by Solow [6] was then used to determine a confidence interval on the cross-validation assignment rate by resampling with replacement and repeating the entire CVA analysis, including the determination of the number of PC axes. The subroutines to determine the optimal numbers of PC axes and estimate this confidence interval are included in the program CVAGen6n [42].

### Dimensionality reduction using partial least squares

In this approach developed by Kemsley [23], the covariance matrix between the measurements for each specimen and an N × S classification matrix is first calculated. The N × S classification matrix has N rows, one for each specimen, and S columns, where there are S groups. Following Kemsley's approach, a specimen receives a score of (N-n_*i*_)/N in the *i*th column of the classification if it is a member of the *i*th class, and a score of -n_*i*_/N in that column if it is not, so that the columns all have a mean of zero. This form of "dummy coding" is often used in multiple regression analysis. A singular value decomposition (as discussed in [53]) of the covariance matrix of the measurements and this classification matrix yields S axes that summarize the greatest pattern of covariance of the measurements with the classification variables, analogous to the way PC axes summarize the patterns of variance.

The scores of the specimens along these SVD axes are then used as variables in the CVA analysis. This approach as a whole is very similar to a multiple regression analysis. Kemsley [23] notes that in fact S-1 columns in the classification matrix would be sufficient to specify the group membership of all individuals (since if a specimen is not in the first S-1 groups, it must be in the last group S). However, Kemsley advocates use of S columns and S SVD axes, which we found to produce higher classification rates than the use of S-1 axes.

## Competing interests

The author(s) declare that they have no competing interests.

## Authors' contributions

HDS designed the study, wrote the IMP series software used in the analysis, performed most of the data analysis, helped draft the figures, and was the lead author of the draft and manuscript. KMC and JMP scanned and digitized feather outlines, performed some data analysis, and assisted in drafting the manuscript and preparing figures. SRM performed dissections, determined specimen ages via dissection and visual inspection of tail feather shape, prepared the final figures, and helped write and revise the manuscript.
